# Supplementary material for: Dual-color fluorescent nanoparticles showing perfect color-specific photoswitching for bioimaging and super-resolution microscopy
Source: Nat Commun. 2019 Jul 12;10:3089. doi: 10.1038/s41467-019-10986-4 (PMC6626011; doi:10.1038/s41467-019-10986-4)
Supplement: Supplementary file 1 — Supplementary Information PDF [file 41467_2019_10986_MOESM1_ESM.pdf]

Supplementary Information for

Dual-Color Fluorescent Nanoparticles Showing Perfect Color-Specific Photoswitching for Bioimaging and Super-Resolution Microscopy

Kim et al.

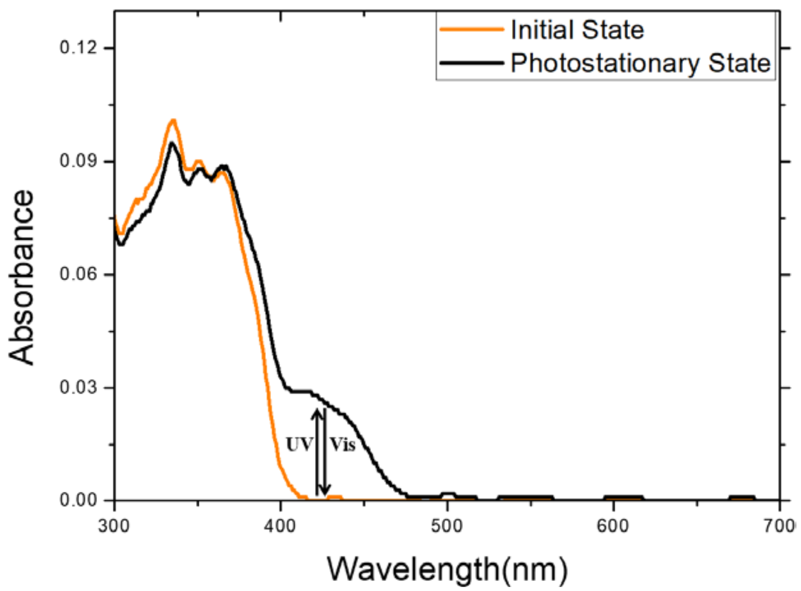

Supplementary Figure 1. UV-vis absorption spectra changes of the mixture film containing DBTEO and HPNIC (DBTEO:HPNIC=1.5 wt%: 1 wt% doped in PMMA) upon UV and visible light irradiations.

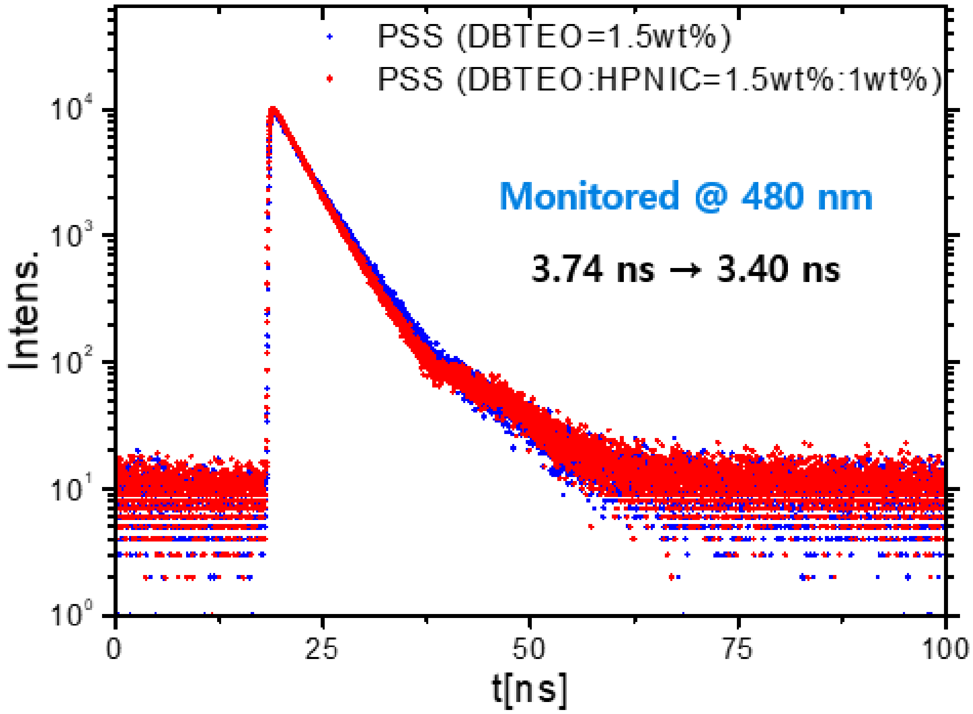

Supplementary Figure 2. Lifetime decay profiles of the emission of closed form DBTEO monitored at 480 nm with 377 nm excitation: (blue dots) DBTEO = 1.5 wt% in PMMA film and (red dots) DBTEO:HPNIC = 1.5 wt%: 1 wt% in PMMA film.

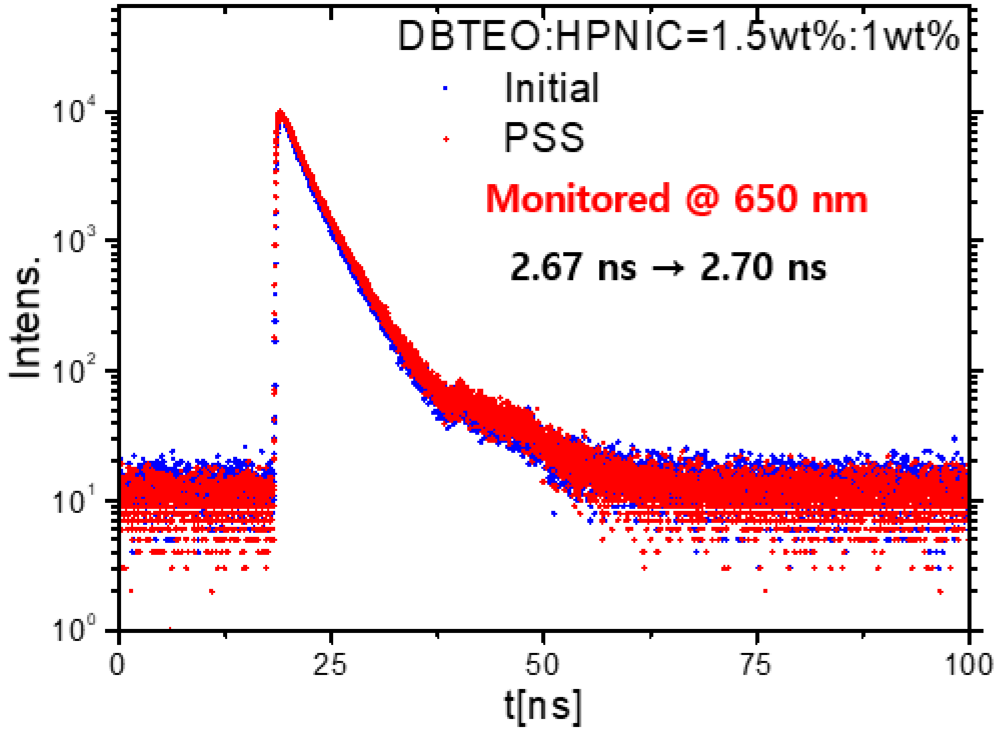

Supplementary Figure 3. Lifetime decay profiles of the emission of HPNIC monitored at 650 nm with 377 nm excitation: (blue dots) initial state and (red dots) photostationary state (PSS) of PMMA film (DBTEO:HPNIC = 1.5 wt%: 1 wt%).

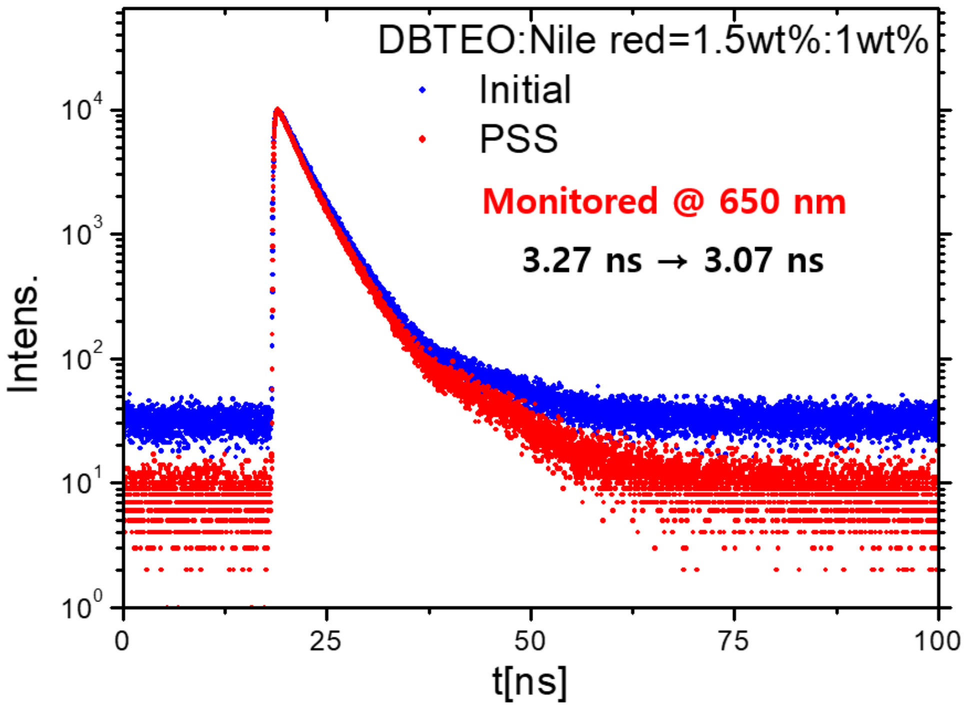

Supplementary Figure 4. Lifetime decay profiles of the emission of Nile Red monitored at 650 nm with 377 nm excitation: (blue dots) initial state and (red dots) PSS state of PMMA film (DBTEO:Nile Red = 1.5 wt%: 1 wt%).

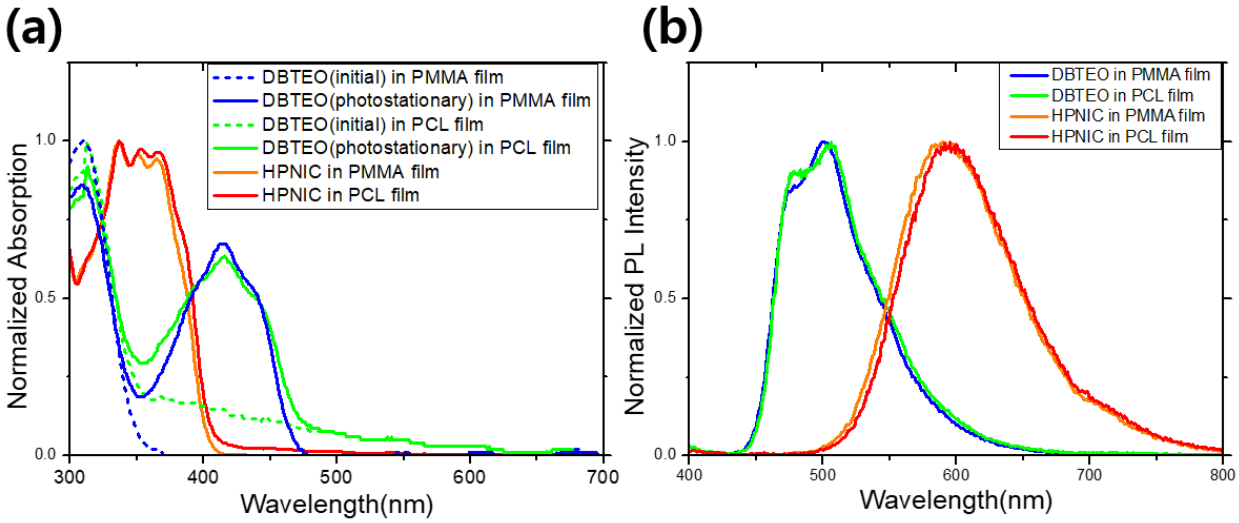

Supplementary Figure 5. (a) Normalized UV-vis absorption spectra and (b) normalized photoluminescence (PL) spectra of DBTEO and HPNIC in PMMA and PCL films.

Supplementary Table 1. Absorption wavelength, fluorescence wavelength, and fluorescence quantum yield of DBTEO and HPNIC in PMMA films, PCL films, and PCL nanoparticles.

	Absorption maximum ($\lambda_{abs,max}$/nm)		Fluorescence maximum ($\lambda_{emi,max}$/nm)		Fluorescence quantum yield (Φ_F)	
	**DBTEO**	**HPNIC**	**DBTEO**	**HPNIC**	**DBTEO**	**HPNIC**
**PMMA film**	**312(o), 415(c)**	**337**	**500**	**590**	**0.55**[a]	**0.11**[a]
**PCL film**	**312(o), 415(c)**	**337**	**504**	**594**	**0.50**[a]	**0.11**[a]
**PCL nanoparticle**	**312(o), 415(c)**	**337**	**504**	**594**	**0.48**[b]	**0.12**[b]

[a]Absolute PL quantum yields were obtained using a QM-40 spectrophotometer equipped with an integrating sphere. [b]Relative PL quantum yields were obtained using fluorescein as a reference dye.

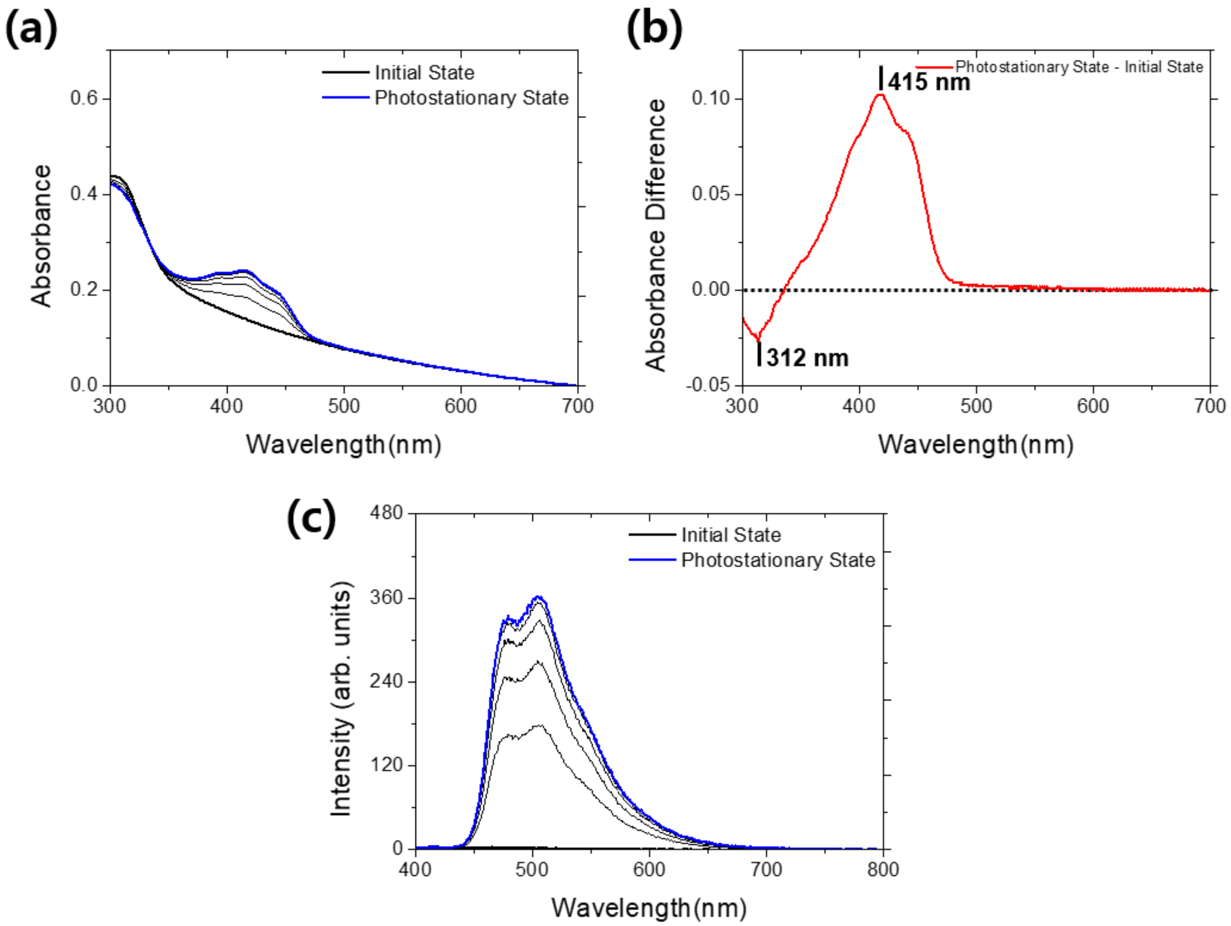

Supplementary Figure 6. (a) UV-vis absorption spectra of the PCL nanoparticles doped with DBTEO (4 wt% to PCL matrix) upon UV and visible light irradiations. (b) Differential between the absorptions of the initial state and photostationary state (PSS). Two wavelengths, 312 nm and 415 nm, are assigned to the absorption maximum wavelengths of open and closed form DBTEO, respectively. (c) Photoluminescence (PL) spectra of DBTEO in PCL nanoparticle (4 wt% to PCL matrix) upon UV and visible light irradiations.

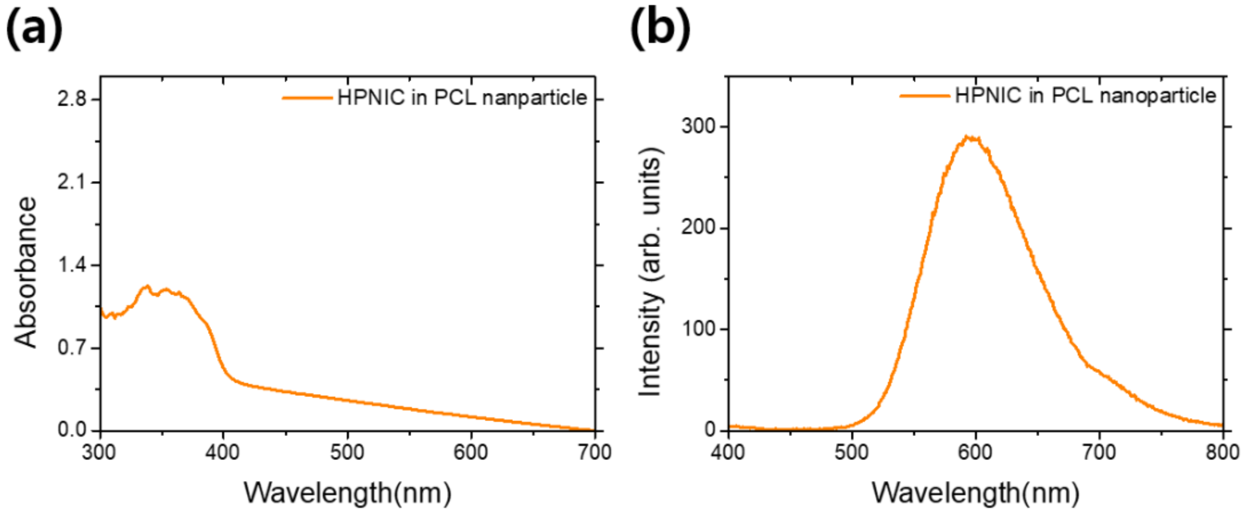

Supplementary Figure 7. (a) UV-vis absorption spectrum and (b) photoluminescence (PL) spectrum of the PCL nanoparticles doped with HPNIC (4 wt% to PCL matrix).

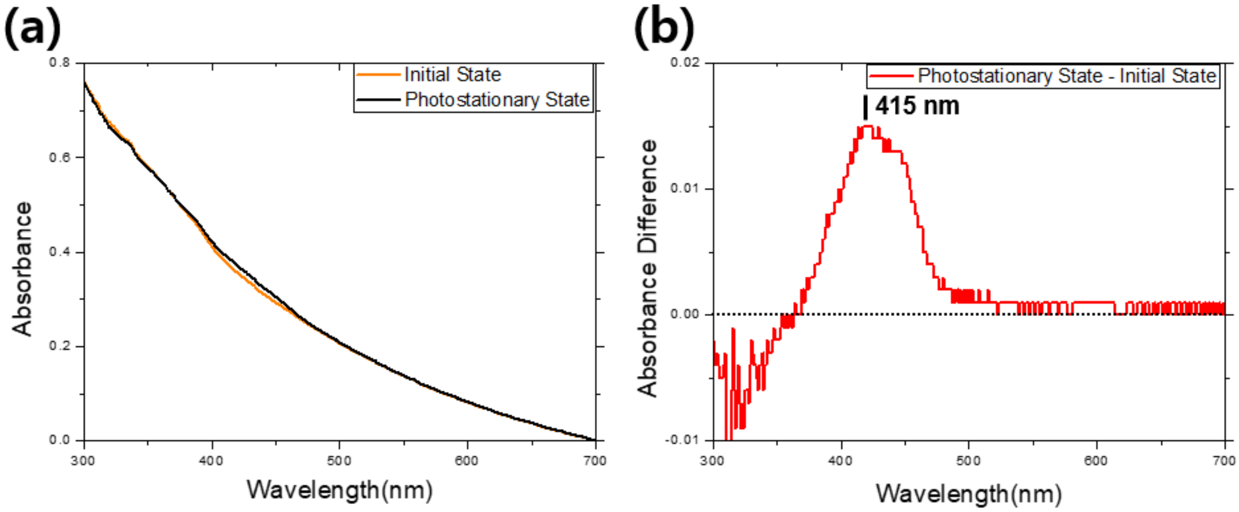

Supplementary Figure 8. (a) UV-vis absorption spectra of the PCL nanoparticles doped with DBTEO and HPNIC (DBTEO:HPNIC=1.5:1 in weight doped in PCL, total concentration of the dyes to PCL matrix is 1 wt%) upon UV and visible light irradiations. (b) Differential between the absorptions of the initial state and photostationary state (PSS). The maximum wavelength is 415 nm, which is identical to the absorption maximum wavelength of closed form DBTEO.

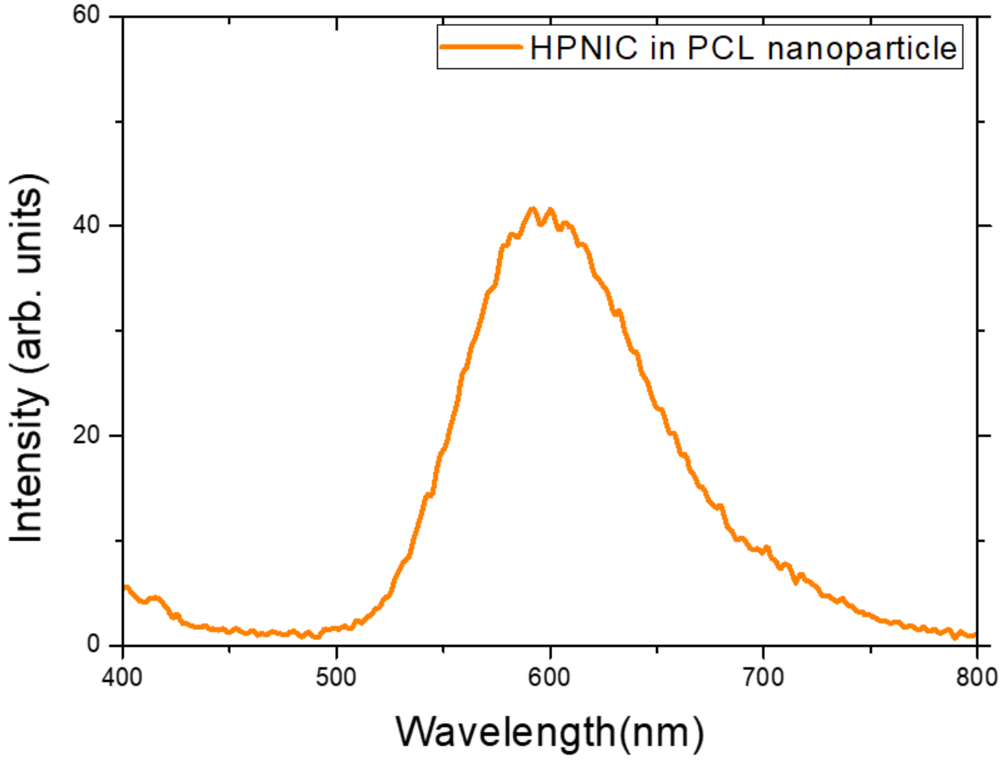

Supplementary Figure 9. Photoluminescence (PL) spectrum of HPNIC in PCL nanoparticle (doping ratio: 0.4 wt% to PCL matrix).

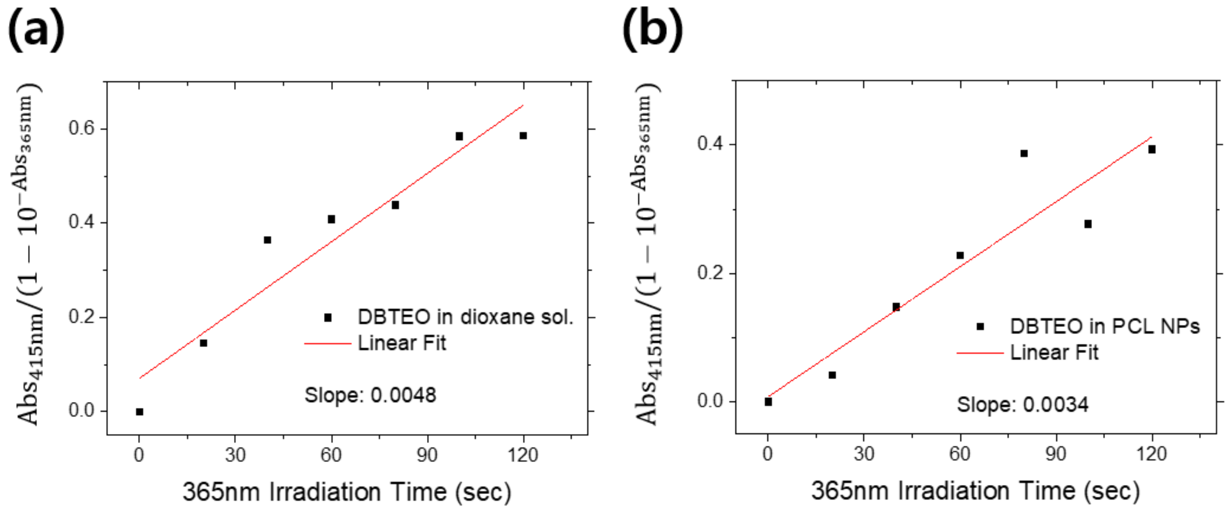

Supplementary Figure 10. Photocyclization quantum yield measurement. (a) Corrected absorption of DBTEO at 415 nm in dioxane solution upon continuous irradiation of 365 nm light. (b) Corrected absorption of DBTEO at 415 nm in PCL nanoparticles upon continuous irradiation of 365 nm light. From the comparison of two slopes in (a) and (b), the photocyclization quantum yield of DBTEO in PCL nanoparticles is calculated from the reference value (0.28) in dioxane solution, resulting in a value of 0.20.[1]

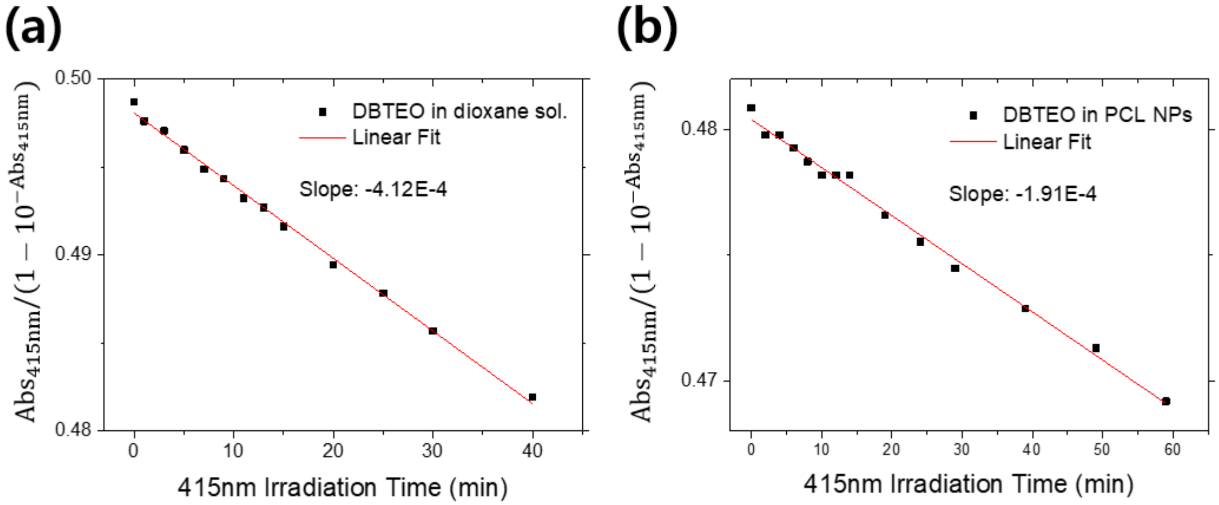

Supplementary Figure 11. Photocycloreversion quantum yield measurement. (a) Corrected absorption of DBTEO at 415 nm in dioxane solution upon continuous irradiation of 415 nm light. (b) Corrected absorption of DBTEO at 415 nm in PCL nanoparticles upon continuous irradiation of 415 nm light. From the comparison of two slopes in (a) and (b), the photocycloreversion quantum yield of DBTEO in PCL nanoparticles is calculated from the reference value (0.18) in dioxane solution, resulting in a value of 0.083.[1]

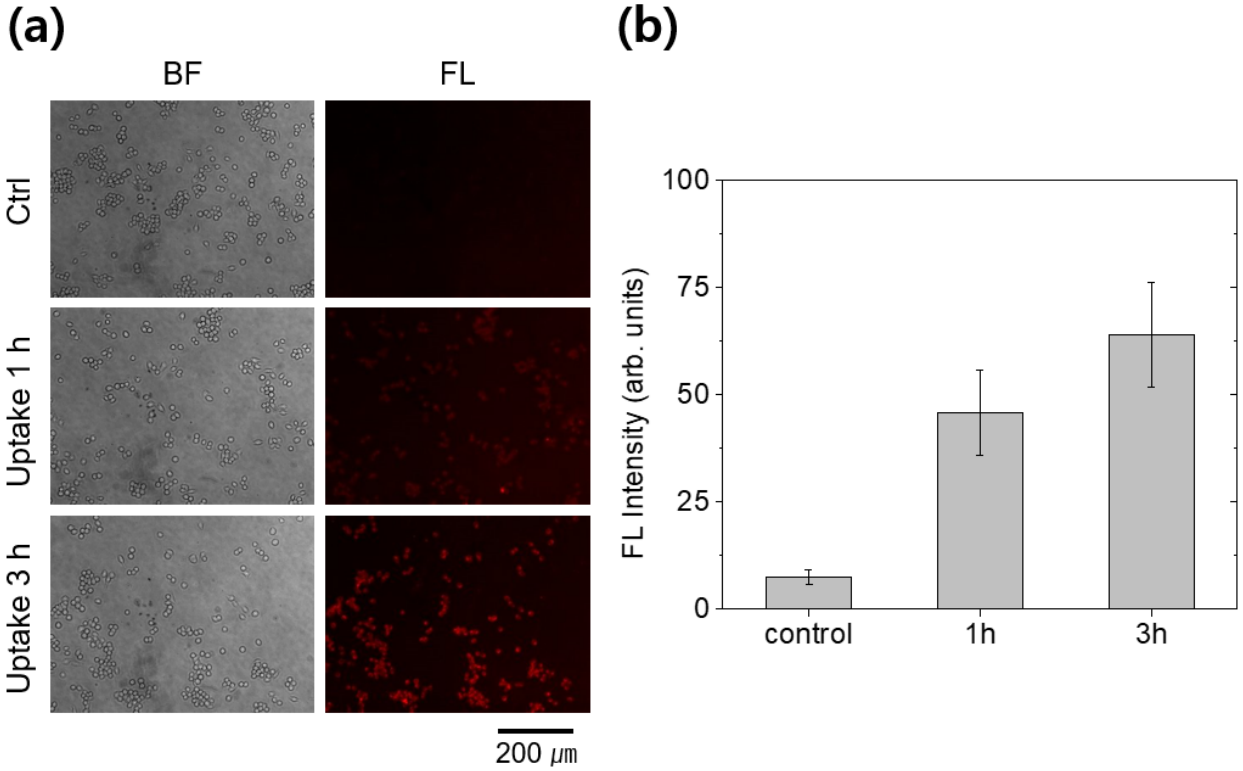

Supplementary Figure 12. (a) Fluorescence images (orange region) of RAW264.7 cells indicating the intracellular delivery of PCL nanoparticles containing DBTEO and HPNIC after 1-3 h incubation. (b) Fluorescence intensity at the cells obtained from the fluorescence images in (a).

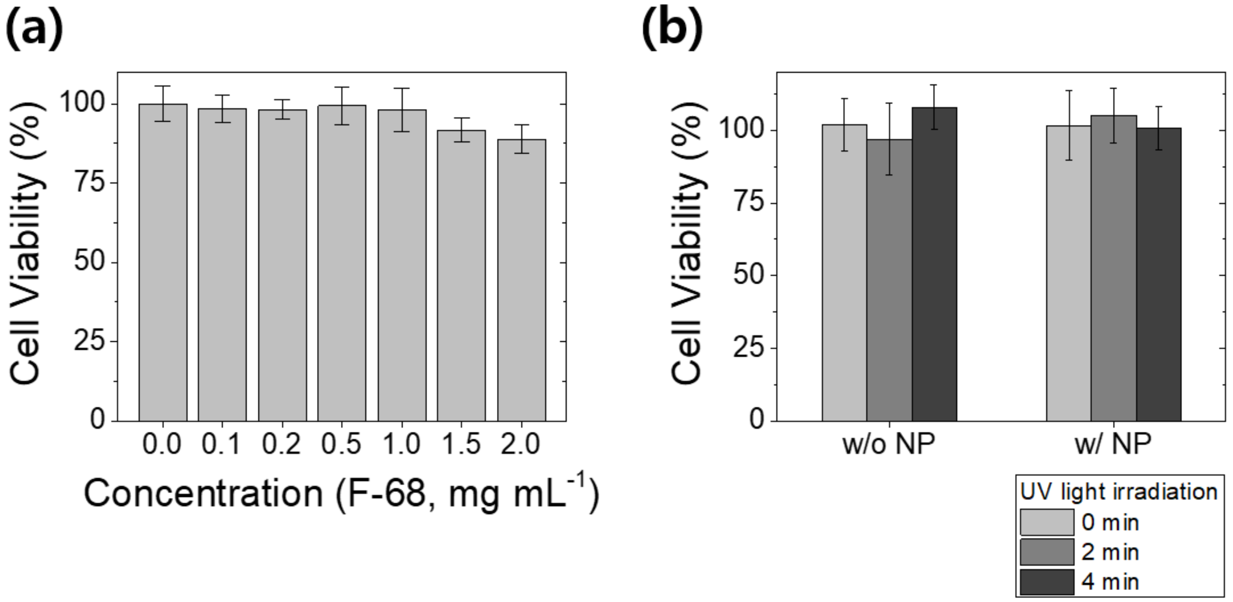

Supplementary Figure 13. (a) In vitro cytotoxicity of Pluronic® F-68 PCL nanoparticles (5 wt% of PCL to F-68) containing DBTEO and HPNIC against RAW264.7. The cells were treated at various concentrations for 1 h. (b) Photo-induced cytotoxicity of the PCL nanoparticles (1 mg mL^{-1} by F-68 concentration) upon exposure to UV-light (365 nm) against RAW264.7 cells. The cell viabilities were evaluated by the colorimetric MTT assay according to the literature procedure.[2]

.

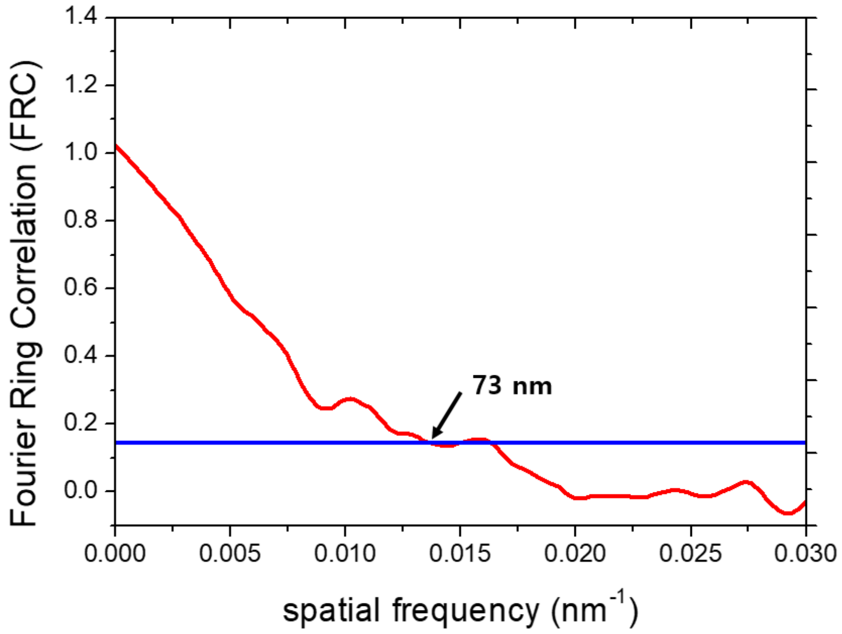

Supplementary Figure 14. Fourier ring correlation (FRC) curve of the localizations presented in Figure 8a: (red line) a smoothed FRC curve and (blue line) a resolution threshold criterion 1/7 (≈ 0.143). The spatial resolution of the super-resolved image is calculated from the intersection between the FRC and the threshold, resulting in a value of about 73 nm.[3]

Supplementary References

1. Uno, K. *et al.* In situ preparation of highly fluorescent dyes upon photoirradiation. *J. Am. Chem. Soc.* **133**, 13558-13564 (2011).

2. Mosmann, T. Rapid colorimetric assay for cellular growth and survival: Application to proliferation and cytotoxicity assays. *J. Immunol. Methods* **65**, 55-63 (1983).

3. Nieuwenhuizen, R. P. *et al.* Measuring image resolution in optical nanoscopy. *Nat. Methods* **10**, 557-562 (2013).
